# Memantine for the Treatment of Behavioral Disturbance in Unspecified Major Neurocognitive Disorder

**DOI:** 10.7759/cureus.17685

**Published:** 2021-09-03

**Authors:** Peyton H Terry, Kareem Seoudy, Meredith S Lee, Keri A Stevenson

**Affiliations:** 1 Psychiatry and Neurobehavioral Sciences, University of Virginia Health System, Charlottesville, USA

**Keywords:** behavioral disturbance, alzheimer’s disease, dementia, major neurocognitive disorder, memantine, alzheimer’s dementia, geriatric psychiatry

## Abstract

In this case report, we aimed to examine how the use of memantine in an elderly gentleman with unspecified major neurocognitive disorder (NCD) led to significant clinical improvement in his behavioral disturbances. After presenting to the psychiatric ward due to aggressive behavior at his assisted living facility, the patient continued to exhibit numerous disruptive and confrontational behaviors while hospitalized. Memantine was started at 5 mg daily with gradual titration up to 10 mg twice daily over the course of four weeks, with marked improvement in behavior as well as an increase in Montreal Cognitive Assessment (MoCA) score by five points after seven weeks of treatment. Given our experience and the safety profile of memantine, we conclude that memantine may have a role in the treatment of behavioral disturbances in patients with unspecified major NCD, though further research will be necessary to define this role.

## Introduction

Major neurocognitive disorder (NCD) currently affects up to 5.7 million people in the United States, with numbers expected to mount up to 65.7 million in 2030 [1]. Unfortunately, behavioral disturbances are exceedingly common in patients with major NCD, representing a significant source of morbidity and mortality in this population. This is particularly burdensome to patients in hospitals and assisted living facilities (ALFs), leading to increased lengths of stay and decreased quality-of-life measures [2]. Furthermore, behavioral disturbance increases psychological stress and fatigue imposed on family and caregivers [3].

Despite the overwhelming consequences of behavioral disturbance in major NCD, we have relatively few options for treatment, many of which lack robust supportive data. Memantine, an N-methyl-D-aspartate (NMDA) antagonist used in moderate-to-severe Alzheimer dementia (AD), has been shown to improve behavioral disturbance in AD patients [4]. In this case report, we aimed to assess the effectiveness of memantine for the treatment of behavioral disturbance in a patient with unspecified major NCD.

## Case presentation

A 76-year-old male with a psychiatric history of bipolar disorder type 1, panic disorder, and dependent personality disorder presented to the emergency department with behavioral disturbance characterized by threatening a staff member at his assisted living facility when he did not receive his food at his preferred time.

According to the staff at his ALF, the patient was compliant with a medication regimen including lamotrigine (50 mg twice daily), clozapine (100 mg nightly), fluvoxamine (50 mg nightly), and clonazepam (0.5 mg twice daily), as well as apixaban for a history of atrial fibrillation. Notably, the patient did not take medication for his essential hypertension due to intermittent orthostatic hypotension. A family member denied a history of neurological disease in the patient or any family history of dementia. During an interview and examination by a psychiatrist, the patient reported no mood symptoms and showed no signs of a manic or depressive episode. Of note, the patient’s vital signs were within normal limits, and initial laboratory studies, including a complete blood count, complete metabolic panel, urinalysis, and urine drug screen, were unremarkable. The patient was admitted to the psychiatric unit for management of his symptoms and initially continued on his home medications.

**Figure 1:**
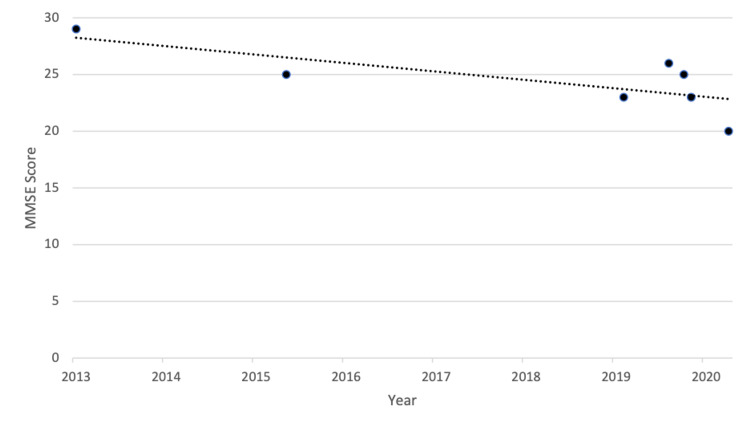
Patient's MMSE scores from 2013 to 2020 MMSE: Mini-Mental State Exam.

Throughout his hospital course, the patient exhibited behavior indicative of major NCD, with an initial Montreal Cognitive Assessment (MoCA) score of 16/30, though the etiology was unclear. For instance, the patient demonstrated a disruptive and confrontational attitude, hyperphagia with excessive carbohydrate consumption, forgetfulness with perseveration on requests for denture cream and Chapstick, and disinhibited sexual commentary, all of which were concerning for frontotemporal dementia. Concomitantly, the patient also exhibited increasing paranoia. However, given the patient’s medical history of hypertension and chronic atrial fibrillation, vascular dementia (VaD) was also a potential cause. A review of the medical record showed the patient had a gradual decline in memory function over time, with Mini-Mental State Exam (MMSE) scores trending from 29/30 to 20/30 over the past seven years based on a review of his electronic medical record at our institution (Figure 1), raising concern for possible AD. The patient refused magnetic resonance imaging.

**Figure 2:**
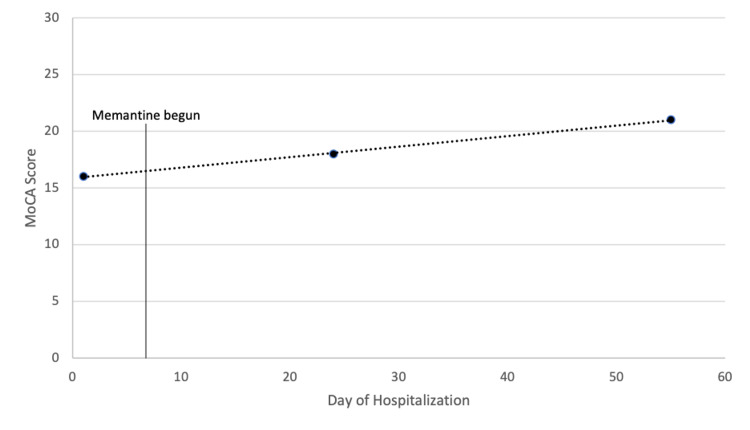
Patient's MoCA scores throughout hospitalization MoCA: Montreal Cognitive Assessment.

Upon admission, the patient’s clonazepam (0.5 mg twice daily) was discontinued indefinitely, and hydroxyzine (25 mg every four hours as needed) was prescribed for anxiety during the duration of hospitalization without clinical improvement in behavioral symptoms; the patient’s other home medications were continued upon admission without alteration. The decision was made to begin therapy with memantine to treat the patient’s continued and unimproved behavioral disturbances. Memantine was started on hospital day six at a dose of 5 mg daily, increasing by 5 mg each week until reaching 20 mg daily (10 mg twice daily). After beginning treatment, many of the patient’s problematic behaviors improved. He exhibited reductions in social isolation, disruptive and disinhibited behavior, intrusiveness, perseverative requests, and paranoia. Serial MoCA tests were administered throughout the memantine treatment regimen, which demonstrated improvement by two points after two weeks of therapy, and by three points after seven weeks of treatment (Figure 2). Of note, after more than three weeks of hospitalization, the patient's fluvoxamine dose was increased to 100 mg nightly, occurring before the final dose increase of memantine. No relapse of behavioral symptoms was noted in the remainder of his four-month hospitalization.

## Discussion

The use of memantine for the treatment of moderate-to-severe AD is well-documented. A 1999 randomized, placebo-controlled trial by Winblad et al. found that memantine improved functional and global outcomes in patients with moderate-to-severe AD [5]. In 2003, a similar study by Reisberg et al. further demonstrated memantine’s efficacy in improving cognition, in addition to function [6]. A third study in 2004 by Tariot et al. comparing memantine and donepezil in moderate-to-severe AD confirmed the findings of previous studies and found significant improvements in behavior with the use of memantine [7].

Nonetheless, the role of memantine in the treatment of mild AD and other forms of major NCD, especially with regard to the improvement of behavioral disturbance, is less established [8]. A meta-analysis in 2007 demonstrated memantine’s efficacy in improving cognition in patients with VaD; however, the study found no significant benefit in function or behavior [9]. Studies investigating memantine’s use in Parkinson’s disease (PD), dementia with Lewy bodies (DLB), frontotemporal dementia (FTD), and HIV-associated neurocognitive disorder (HAND) have shown mixed results [10-14].

A 2019 Cochrane meta-analysis on memantine’s role in dementia treatment reported moderate-certainty evidence that memantine had no significant benefit in mild AD, and it further described low- or very low-certainty evidence for the use of memantine in other dementia etiologies (e.g., PD, DLB, FTD, and HAND), likely due in part to the small sample sizes of the studies [15]. However, the Cochrane review did find low- to moderate-certainty evidence for modest improvement in cognition and behavior in patients with mild-to-moderate VaD treated with memantine [15]. These results indicate that a link between memantine and improvement in the behavioral disturbance in patients with major NCD due to etiologies other than AD may be elucidated with further trials in these patient populations. However, it is inherently challenging to study "unspecified" major NCD due to the heterogeneous symptomatology and likely multifactorial nature of the disease in these patients.

Also of interest in our case is the clinically significant improvement in the patient’s paranoia after beginning treatment with memantine. One previous case series reported improvement of symptoms in patients with schizophrenia who are treated with memantine in addition to their pre-existing antipsychotic regimen, though the improvements were found primarily in negative symptoms [16]. A 2016 randomized controlled trial investigating the addition of memantine to patients with schizophrenia being treated with varying doses of clozapine also found improvement in negative symptoms [17]. In a one-year continuation study of memantine treatment, Veerman et al. found that both positive and negative symptoms of schizophrenia improved [18].

If memantine is to have a role in the treatment of behavioral disturbance in unspecified major NCD, its potential benefits must outweigh the risks of treatment. Studies have generally shown that memantine has no increased risk of adverse events compared to placebo, and expert consensus supports the safety of memantine [19-20]. The same 2019 Cochrane meta-analysis reported no difference in the risk of developing an adverse event between patients receiving memantine versus placebo; furthermore, this risk did not vary according to the etiology of major NCD [15]. Of all adverse events reported while taking memantine, those found to be significantly more common in patients taking memantine were dizziness and headache. However, the meta-analysis found no significant difference between memantine and placebo in risk of falls of particular importance in the elderly population [15].

This particular study has a number of limitations, including its nature as a single case study, the constraints of currently available cognitive testing, and the challenge of isolating the effects of a single drug in the complex clinical care of a psychiatric patient. Nonetheless, the substantial benefit seen with the use of memantine in this patient with likely multifactorial etiologies of major NCD was clinically remarkable and warrants further investigation.

## Conclusions

Memantine may have the potential to improve behavioral disturbances in patients with major NCD apart from purely AD. Furthermore, the clinically significant improvement in this patient’s paranoia raises the question of whether memantine may have a direct effect on paranoia or whether it may augment the potency of clozapine. Given the observed efficacy in this clinical case as well as the medication’s favorable side-effect profile and safety in the elderly population, memantine warrants further exploration as a potential therapy to improve behavioral disturbance in patients with varied or multifactorial etiologies of major NCD.
